# Detecting the Mechanism of Action of Antimicrobial Peptides by Using Microscopic Detection Techniques

**DOI:** 10.3390/foods11182809

**Published:** 2022-09-12

**Authors:** Muhammad Zohaib Aslam, Shumaila Firdos, Zhousi Li, Xiang Wang, Yangtai Liu, Xiaojie Qin, Shuo Yang, Yue Ma, Xuejuan Xia, Bolin Zhang, Qingli Dong

**Affiliations:** 1 School of Health Sciences and Engineering, The University of Shanghai for Science and Technology, Shanghai 200093, China; 2 Dera Ghazi Khan Section of Punjab Livestock and Dairy Development Department, Dera Ghazi Khan 32200, Pakistan; 3 Beijing Key Laboratory of Forest Food Processing and Safety, Beijing Forestry University, Beijing 100083, China

**Keywords:** antimicrobial peptides, whey protein, pathogenic bacteria, microscopic techniques, recent developments

## Abstract

Increasing antibiotic resistance has shifted researchers’ focus to antimicrobial peptides (AMPs) as alternatives to antibiotics. AMPs are small, positively charged, amphipathic peptides with secondary helical structures. They have the ability to disrupt the bacterial membrane and create wedges due to electrostatic differences. Water molecules enter the pathogens through those wedges and disrupt their normal cellular functioning, eventually causing the death of the pathogens. Keeping in mind the importance of AMPs, this review compiles recent data and is divided into three parts. The first part explains the AMP structure and properties, the second part comprises the spectroscopy techniques currently used for evaluating the AMP-bacterial targeting mechanism as well as its structure and safety; and the third part describes the production of AMPs from an animal source (whey protein). Most of the peptides that were used in recent studies have been either the precursors of a natural peptide or synthetic peptides with some modifications, but data on the exploitation of dairy protein are scarce. Among the little-studied milk proteins and peptides, in the last three years, whey protein has been studied the least based on the reported data. Because whey protein is a leftover part of cheese making that often drains out as cheese waste, causing soil and environmental pollution, today, the need of the hour is to produce safe AMPs from whey protein. The use of whey protein that is based on hydrolyzing lactic acid bacteria with some structural modifications can increase AMPs’ potency, stability, and safety, and it can also help to avoid soil and environmental pollution as a result of whey drainage.

## 1. Introduction

Increasing antibiotic resistance (ABR) poses a serious threat to human and animal populations in the form of foodborne diseases. To address the issue of antibiotic resistance due to the misuse of antibiotics, the WHO (World Health Organization, Geneva, Switzerland) and the FAO (Food and Agriculture Organization, Rome, Italy) launched the “Global Action Plan to Combat Antimicrobial Resistance” in 2015. The European Union, being the largest food importer, has banned the use of antimicrobial growth promoters [1]. Because of the misuse of antibiotics, pathogenic bacteria have developed drug-resistant genes and show resistance to antimicrobial drugs. This bacterial resistance results in great damage to the economy and to our health in the form of food spoilage and health issues in animal and human populations. It is now important to find alternatives to antibiotics that can minimize this damage to health and the effects of many foodborne diseases [2].

To combat ABR, proteins are the next potent class that need to be explored to determine their antimicrobial activity. Protein and its peptides have the ability to be used as safe food supplements with many health benefits, including nutraceuticals and AMPs. AMPs are a major part of innate immunity and are found in every organism, serving as antimicrobial agents according to their structure and nature. AMPs are quite different and are specific in targeting pathogenic cells via disrupting the cell membranes as compared to conventional antibiotics, which only target the receptor protein to stop the cell growth [3].

AMPs are described as combinations of amino acid sequences that have a lower tendency than antibiotics to develop resistant genes in pathogens if they are used as food additives or prophylactic measures [4]. Many studies have reported the usefulness of bioactive peptides as AMPs due to their precise targeting of bacterial cell membranes. The main components of AMPs include their molecular length, hydrophobicity, net charge, and secondary structure, which is important for targeting pathogenic bacterial cells.

Hydrophobicity is the main characteristic of AMPs that affects their cell permeability. Hydrophobic amino acid residues such as valine, leucine, isoleucine, alanine, methionine, phenylalanine, tyrosine, and tryptophan sequence are indicators of the peptide’s hydrophobicity [5]. AMPs mainly target pathogens via receptor- and non-receptor-mediated pathways. In the receptor-mediated pathway, peptides target the protein receptors on the cell membrane and affect the membrane homeostasis, while in the non-receptor-mediated pathway, peptides directly target the cell membrane and penetrate via the helical structure to disrupt the cell. [6] Because of their effective roles in targeting pathogenic bacterial cells, researchers are trying to explore new sources for developing AMPs. Approximately three thousand AMPs have been developed and identified so far, but only six of them are approved by the United States Food and Drug Administration for use as an alternative to drugs because most AMPs are still in the research phase [7]. They are prepared using different techniques such as solvent extraction, enzymatic hydrolysis, fermentation, and genetic engineering. Most of the previous research has been conducted on the use of enzymatic hydrolysis and fermentation techniques to produce AMPs [8].

Milk whey, which is considered cheese waste, is a rich source of bioactive peptides and essential and branched-chain amino acids that can be used as food supplements with many health-promoting and regulating benefits [9]. Due to its rich protein contents, whey protein is a potential candidate for producing AMPs.

**Figure 1:**
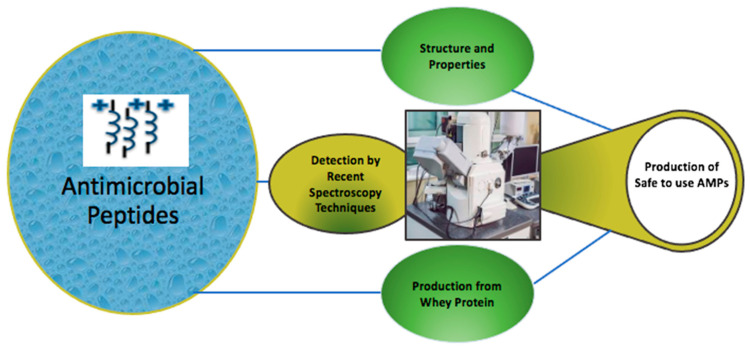
The production of safe AMPs.

This study is designed to highlight the structure, properties, targeting mechanism, and recent developments in the production of AMPs, as well as to validate the use of whey to produce AMPs as shown in Figure 1.

## 2. Structure and Properties of AMPs

AMPs are a class of small bioactive peptides that exist in nature and comprise a vital part of the innate immune systems of different organisms [10].

Three thousand AMPs have been identified from different sources so far, but only six are approved by the United States Food and Drug Administration (Silver Spring, MD, USA) [11]. Over three thousand peptides are listed in the United States database. All the peptides in the database have a different structure, amino acid sequence, length, and charge. The majority of the listed peptides are derived from frogs and considered small peptides with a length of less than 51 amino acids. Most of these peptides are hydrophobic in nature and cationic amphipathic with a net positive charge. The approved peptides have the quality of targeting the bacterial cell by receptor binding, biological pathway inhibition, and membrane penetration [12].

Wang’s laboratory in China has also developed a database of AMPs and divided those peptides according to their source and secondary structure. According to that database, 74.1% of peptides were extracted from animal sources, and 11.2%, 11.1%, 0.6%, 0.2%, 0.2%, and 2.5% were extracted from plants, bacteria, fungi, protists, archaea, and synthetic sources, respectively. Peptide categorization according to the secondary structure is based on the alpha and beta structure, and 67.2% of AMPs have an alpha structure; another 17.3% have alpha-beta structures, 12.7% have beta structures, and 2.8% have non-alpha-beta secondary structures. Environmental factors such as salt and PH levels affect the normal efficiency of AMPs. Recently, some techniques such as the modification of the amino acid sequences and PH modulation of peptides have been considered useful in order for AMPs to function normally in the presence of high-salt or high-PH environments [13].

Bioactive peptides in the form of antimicrobial peptides (AMPs) contain diverse amino acid sequences (smaller peptides with 10–100 amino acids) with a net positive charge (+2 to +9) and cationic amphipathic characteristics [14]. The definition of AMPs is updated according to new discoveries, e.g., peptides 2–100 amino acids long with or without a charge are categorized as AMPs. These peptides are prepared from either a natural source, a synthetic source, or a combination of both [15].

The amino acid constituents, molecular length, net charge, hydrophobicity, and secondary structures of the peptides are the main factors that affect the properties of AMPs.

The amino acid sequence of an AMP is the main point that determines its antimicrobial characteristics. AMPs can be either cationic or anionic in nature, but most of the documented AMPs are cationic in nature with a net positive charge. Cationic AMPs contain a sequence of hydrophobic amino acids on one side and of hydrophilic acids on the other [16]. Most cationic AMPs have a general structure including linear cationic α-helical peptides, linear cationic peptides with proline and arginine, and β-sheet peptides with cysteine residues forming disulfide linkages with other peptides. The secondary structures that comprise α-helical and β-sheet peptides are important in targeting pathogens.

Upon storage in an aqueous solution, α-helical peptides such as magainin show no distinct structure, but after interacting with the bacterial membrane, they fold themselves into a helical structure and enter the cell membrane. Meanwhile, AMPs with β-sheet secondary structures such as lactoferricin (lactoferricin is a fragment from the release of lactoferrin following the hydrolysis by pepsin of this whey protein) maintain their original structure in the aqueous solution as well as upon targeting the bacterial membrane [17,18]. There are also 196 anionic and 189 neutral AMPs stored in the AMP directory of the United States database. Amino acid constituents and side-chain arrangement play an important role in the antimicrobial nature of peptides. Proline, arginine, cysteine, and glycine-rich chains of amino acids [19] without any specific 3D structure represent the main constituents of AMPs [20].

## 3. The Mechanism of Targeting the Bacterial Cell

**Figure 2:**
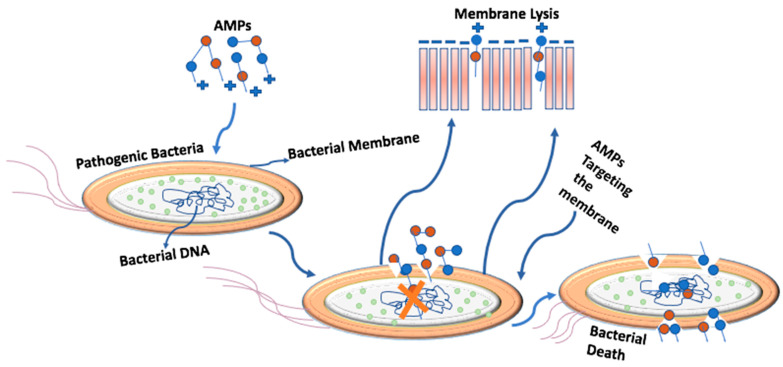
Shows the electrostatic interaction of membranes. Cationic AMPs target the anionic bacterial membrane. AMPs penetrate into the cell membrane and cause cell death.

AMPs approach the target bacterial cell by means of two possible mechanisms: the first mechanism being through the electrostatic interaction between the cationic nature of peptides and the anionic bacterial membrane; the second mechanism being the hydrophobic interaction between the amphipathic nature of peptides and the acyl chains of the lipids on the bacterial membranes. Most research work on this topic has focused on the electrostatic interactions of the membranes, as shown in Figure 2. In this mechanism, after targeting the bacterial membranes, the peptides attach to the target cell because of the electrostatic charge between the peptides and the bacterial membrane. This targeting of the bacterial membrane can occur in many different ways according to the membrane’s mode of action, e.g., carpet mode, detergent mode, barrel-stave mode, toroidal mode, or non-membranolytic mode.

In carpet mode, several peptides accumulate parallel to the target cell, attach to the lipid bilayer, and form pores on the surface; water molecules then enter those pores, eventually causing bacterial cell distortion and death. Peptides with a net positive charge such as cathelicidin LL-37 (sourced from human epithelial cells) target the pathogen in this way [21]. In detergent mode, cationic peptides directly target anionic bacterial cell membranes and form nano-pores on the lipid surface that cause cell lysis.

AMPs of the temporin family (Temporin-She) attack the target pathogen in detergent mode and disrupt the membrane [12]. In the barrel-stave model, peptides accumulate vertically against the lipid layer and form pores with the help of a peptide–peptide interaction. In this mechanism, peptides’ hydrophobic components attach to the target membrane and cause cell lysis. Alamethicin, extracted from fungus, targets pathogenic cells in this way [22]. In toroidal mode, the main groups of peptides and lipid layers are attached to each other, and the peptide and the phospholipid head groups form pores that eventually cause cell death. The main difference between the barrel-stave model and toroidal mode is the positioning of the lipid bilayer membrane; in toroidal mode, the amphipathic side of the lipid bilayer is damaged, while it remains undamaged in the barrel-stave model. AMPs such as PG-1 (porcine protegrin-1) target the bacterial membrane in toroidal mode, whereas in non-membranolytic mode, the AMPs interact with the protein receptors of the target and penetrate the cell to stop protein production and disrupt the metabolic processes that eventually cause cell death. PrAMP, extracted from insects, targets pathogenic cells by inhibiting protein synthesis [23,24]. In all these targeting modes, AMPs’ attachment modes are similar, with slight differences in the peptides’ assembly and attachment around the bacterial lipid bilayers. The majority of the peptides are attached to the lipid bilayer in carpet and toroidal mode.

## 4. Recently Tested Peptides

To date, AMPs have been purified and produced from different origins. Scientists have documented their efficiency in different in vitro and in vivo models. In most of the studies (summarized in Table 1), small peptides such as pyrrhocoricin, magainin 2, alamethicin, porcine protegrin-1, aurein 1.2, human beta-defensin 2, cathelicidin LL-37, nisin, indolicidin, temporin-She, Pro10-1D, AP-64, LL-14, protein-glutamine gamma-glutamyltransferase 2, and crustin were evaluated for their efficacy against pathogenic bacteria. Some of these AMPs, such as alamethicin, porcine protegrin-1, indolicidin, temporin-She, and AP-64, are considered cytotoxic for human use, while the rest of the AMPs (magainin 2, HBD2, LL-37, nisin, Pro10-1D, and protein-glutamine gamma-glutamyltransferase 2) are considered less toxic and safe to use. Among all these safe-to-use AMPs, nisin is used extensively in clinical trials [25,26]. More in vitro and in vivo clinical trials are required to further explore its safety.

Pyrrhocoricin, a proline-rich AMP (PrAMP), has an affinity for DnaK protein in *Escherichia coli*. This peptide does not affect DnaK-deficient *Escherichia coli* but targets the valid type, which is pathogenic in general. Pyrrhocoricin is derived from insects and is considered to be efficient for targeting pathogenic bacteria by binding the stress inducer translational DnaK protein [27]. Magainin 2 targets the lipid bilayer of Gram-positive and Gram-negative bacteria and forms holes on the bacterial membranes that cause the death of the pathogen. It is derived from the skin of the African clawed frog, *Xenopus laevis*. [28]. Human beta-defensin 2, smaller in size and with a low molecular weight (4.3 Da), has been documented to be safe to use as an AMP. It is derived from human skin, and it targets the bacterial membrane, blocking the biofilm formation of pathogens [29]. Cathelicidin LL-37 is a small (37 amino acid) AMP with a low molecular weight (18 kDa) that is derived from epithelial cells. It targets both the cationic and anionic membranes of pathogens and is safe to use [30]. Nisin is safe to use and has been approved by the United States Food and Drug Administration. It is a lantibiotic AMP (derived from the unique amino acid lanthionine) derived from *Lactococcus lactis* [31]. Pro10-1D, a small AMP (10 amino acids) with a low molecular weight (1.4 kDa), was designed in the laboratory. After being evaluated in vitro and in vivo, it is considered safe to use. It directly targets the membrane and damages the bacterial cell [11]. Further research is needed into the safety and stability of these synthetic and natural peptides in order to prove them potent AMPs for use as drugs of choice or food supplements. More attention should be paid in future research to the production of AMPs from a food source such as whey protein, which is a good source of protein and bioactive peptides.

**Table 1:** The source, safety, structure, and action of AMPs.

Name	Source	Secondary Structure	Pathogenic Bacteria	Toxicity	Efficiency	Reference
Monomeric peptide MG2 (Magainin 2)	Skin of African clawed frog	Alpha-helical	*Escherichia coli*	Low toxicity	Targets the membrane and forms toroidal pores	[28]
Peptaibol peptide-Alamethicin	fungus	Alpha-helical	___	Toxic	Targets bi-layer lipid membranes by forming pores	[32]
Cationic AMP PG-1 (Porcine protegrin-1)	porcine neutrophils	Beta-hairpin	Broad spectrum pathogens	Cytotoxic	Targets the membrane by forming pores	[26]
Aurein 1.2 (GLFDIIKKIAESF-NH_2_)	Skin of Australian bell frogs	Alpha-helical	___	___	Targets the bacterial membrane	[33]
Cysteine-rich HBD2 (Human beta-defensin 2)	Human skin	Beta-strand and Alpha-helical	*Pseudomonas aeruginosa*	Non-toxic	Targets the bacterial membrane and inhibits biofilm formation	[29]
Cathelicidin LL-37	Human skin, epithelial cells	Cationic amphipathic Alpha-helical	Broad spectrum pathogens	Non-Toxic	Targets the Cationic and anionic membranes of pathogens	[30]
Nisin	*Lactococcus lactis*	Looped	*Streptococcus mutans*	Non-toxic	Targets the pathogen’s lipid bi-layer membrane	[34]
45 analogs of AMP indolicidin	Neutrophil blood cells of cows	Alpha-helical	Gram^+^ and gram^−^ bacteria	Toxic at high concentration	___	[35]
AMPs of the temporin family (Temporin-She)	The skin of the Sahara Frog	Non-amphipathic α-helical peptide α-helical peptide	*Leishmania infantum,* and *Staphylococcus aureus*	Toxic	Targets Gram^+^ and gram^−^ bacteria by damaging the lipid chain of the membrane	[36]
Pro10-1D	Designed in the lab	Alpha-helical	*Escherichia coli*, and *Acinetobacter baumannii*	Non-toxic	Targets the bacterial membrane and damages the cell efficiently	[11]
AP-64	Human	Alpha-helical	*Escherichia coli* DH5α, *Escherichia coli* O157:H7, *Vibrio cholerae*, and *Pseudomonas aeruginosa*	Cytotoxic	Targets the membrane	[37]
LL-14	lysosomes and polymorphonuclear leukocytes	Helical	*Escherichia coli*, *Salmonella typhi*, *klebsiella* *pneumoniae*, *Staphylococcus aureus*	LL-14 Non-toxic	Membrane depolarization and cell death	[38]
Protein-glutamine gamma-glutamyltransferase 2	Hemoglobin of blood clam	Alpha helical	*Escherichia coli*	Less Toxic	Targets membrane by making nano pores through which cellular material leaks out	[39]
Crustin (rCrus1)	shrimp	Alpha helical	Gram^+^ bacteria	___	Damages the cellular machinery in target cells	[40]

## 5. Recent Developments in the Use of Microscopy Techniques to Highlight AMP Targeting

Recent data related to bacterial targeting mechanisms are compiled and discussed in this section. A wide range of microscopy techniques—such as transmission electron, scanning electron, atomic force, fluorescence, field emission scanning electron, confocal laser scanning, real-time fluorescence, and helium ion microscopy—were used to evaluate the bacterial targeting mechanism against a variety of Gram-positive and Gram-negative bacteria: *Escherichia coli*; *Acinetobacter baumannii*; *Vibrio cholerae*; *Klebsiella pneumoniae*; *Cutibacterium acnes*; *Porphyromonas gingivalis*. In addition, different strains of *Staphylococcus*, *pseudomonas*, *Mycobacterium*, *Salmonella*, and *Enterococcus* were evaluated (as shown in Table 2).

Among all these microscopy techniques, scanning electron, transmission electron, high-speed atomic force, and confocal microscopy are widely used to elaborate the AMP targeting mechanism. Most of the studies reviewed used *Escherichia coli*, *Staphylococcus aureus*, and *Pseudomonas aeruginosa* as the target bacteria because these bacteria account for most foodborne diseases and clinical infections.

The identified targeting mechanism is that AMPs target the pathogenic microbial cell membrane and form nanopores to enter the cellular machinery. After penetrating the cell, AMPs stop the normal metabolic process, which eventually causes cell death. Scientists have documented the AMP targeting mechanism using these techniques for observation, but they are still unable to explain the exact mechanism of how AMPs target and destroy pathogenic bacterial cells; the current suggested mechanism is still based on observation rather than concrete proof. Recent and relevant information is documented in the table below to explain the different techniques used and observations made.

**Table 2:** Provides the necessary information on Synthesized and purified AMPs and pathogenic bacterial strains in order to explain the targeting mechanism. Different microscopy techniques were used and the observations in these recent studies were almost similar.

Pathogens	AMPs	Microscopic Technique	Observations	Reference
*Pseudomonas Aeruginosa*	DP7, (synthetic)	Gel retardation assay	DP7 targets the membrane protein and damages bacterial membrane	[41]
*Staphylococcus aureus*	Porcine beta defensin 2, (synthetic)	Transmission electron microscopy	AMP targets the cell membrane and then enters the cytoplasm	[42]
*Staphylococcus aureus*	Temporin-She, (extracted from frog, mildly cationic with charge of +2)	Scanning electron microscopy	AMP targets the anionic cell membrane	[36]
*Escherichia coli*, and *Acinetobacter baumannii*	Pro10-1D (synthetic with charge of +4)	Scanning electron microscopy	Targets the bacterial membrane	[11]
*Escherichia coli*	P6.2 (synthetic)	Atomic force microscopy	Targets the pathogen at the membrane level	[43]
Methicillin-resistant *Staphylococcus aureus*	PVP (synthetic)	Fluorescence microscopy	Increasse membrane permeability and causes cell lysis	[44]
*Escherichia coli*	Temporin L (extracted from frog skin)	Transmission electron microscopy	After interacting with the membrane protein, this AMP forms nanopores	[23]
*Staphylococcus aureus* and *Staphylococcus epidermidis*.	Cecropin, Magainin 2, and melittin	Field Emission Scanning Electron Microscopy	These AMPs target the membrane and form pores on it	[45]
*Pseudomonas aeruginosa*	undecapeptides (AMP21-24)	Field emission scanning electron microscopy	Targets the membrane	[38]
*Pseudomonas fluorescens*	Temporin-L (extracted from frog)	Confocal Laser Scanning Microscopy	Targets the bacterial biofilm	[46]
*Escherichia coli*	cecropin A (extracted from honeycomb moth)	Scanning electron microscopy	Disrupts bacterial membrane and targets the biofilm	[47]
*Mycobacterium smegmatis*, and *Mycobacterium tuberculosis*	HHC-8, and MM-10 (Synthetic)	Scanning electron microscopy	Targets the membrane and makes it permeable to penetrate	[48]
*Escherichia coli*	*Pa*-Methionine aminopeptidase 2 and *Pa*-Methionine aminopeptidase 2 1.9 (Synthetic)	Atomic force microscopy	Cationic AMPs target the anionic membrane and cause cell death	[49]
Gram^+^ bacteria	rCrus1 (Extracted from shrimp)	Scanning electron microscope and Transmission electron microscope	This AMP causes membrane leakage and structure damage In the pathogen	[40]
*Escherichia coli*	Protamine (extracted from salmon sperm) and OH-CATH-30 (Synthesized)	Electron Microscopy	AMP adheres to the target membrane	[50]
*Escherichia coli* DH5α, *Escherichia coli* O157:H7, *Vibrio cholerae*, and *Pseudomonas aeruginosa*	AP-64 (extracted from human lacking cysteine)	scanning electron microscopy	Targets the membrane	[37]
*Salmonella typhi* TY2	LL-14, VV-14 and ββ-14 (synthetic)	Field emission scanning electron microscopy	Targets the membrane, causes depolarization and eventually cell lysis	[51]
*Escherichia coli*	Protein-glutamine gamma-glutamyltransferase 2 (extracted from blood clam hemoglobin)	Transmission electron microscopy	Increases membrane permeability	[39]
*Escherichia coli*	Disulfide-rich β-defensin AvBD103b (extracted from avian defensin)	Real time Fluorescence microscopy	Targets the outer and cytoplasm membrane and disrupts homeostasis	[52]
*Enterococcus faecalis*, *Klebsiella pneumoniae*, and *Pseudomonas aeruginosa*	Synoeca-MP (extracted from the venom of Synoeca surinama)	Atomic force microscopy	Targets the membrane	[53]
*Eschericia coli* and *Staphylococcus aureus*	Arginine-rich peptide Bac8c^2,5Leu^ (synthetic)	Scanning electron microscopy	Effective in targeting pathogens	[54]
*Enterococcus hirae*	SAAP-148 (synthetic)	Fluorescence Microscopy	Disrupts the Anionic membrane and cell shrinkage	[55]
*Porphyromonas gingivalis*	DP7 (synthetic)	Transmission electron microscopy	Targets the bacterial membrane and inhibits biofilm formation	[56]
Nosocomial bacterial pathogens	Pardaxin, MSI-78, dermaseptin-PC, and Cecropin B (Synthetic)	Helium ion microscopy	Targets the membrane	[57]
*Streptococcus agalactiae*	NZX and P2 (extracted from fungal defensin)	Scanning electron microscopy	Targets the cell wall and disrupts the membrane	[58]
*Bacillus circulans*	BaCf3 (extracted from *Bacillus amyloliquefaciens*)	Scanning electron microscopy	Targets the membrane by pore formation	[59]
*Staphylococcus aureus, Streptococcus agalactiae,Vibrio harveyi*, *Vibrio alginolyticus*, *Escherichia coli*, and *Edwardsiella tarda*	TroNKL-27 (extracted from golden pompano)	Scanning electron microscopy	Targets the pathogen and degrades the DNA after penetrating	[60]
*Staphylococcus aureus*	Cruzioseptins (extracted from splendid treefrog)	Fluorescence Microscopy	Targets the bacterial membrane	[61]
Methicillin-resistant *Staphylococcus aureus*, *Escherichia coli*	Proline-rich antimicrobial peptides	Scanning electron microscopy	Efficient in targeting the membrane	[62]
*Staphylococcus aureus*	LCMHC (extracted from Larimichthys crocea)	Transmission electron microscopy	Targets the cell membrane	[63]
*Cutibacterium acnes*	WSKK11 and WSRR11	Scanning electron microscopy, transmission electron microscopy	Efficient in targeting the pathogens	[64]

## 6. The Production of AMPs from Whey Protein

AMPs are protein precursors and can be purified and designed according to their specific amino acid sequence. These proteins are part of either plant or animal proteins. AMPs can be produced by using physical, chemical, and biological techniques. Proteins are heat-treated in the physical techniques, acidic and alkaline catalysts are used to hydrolyze protein in the chemical techniques, and enzymes and fermentation are used in the biological techniques to hydrolyze the AMPs into smaller peptides. Biological techniques are considered safe and can achieve the desired results. We focus on biological techniques, using lactic acid bacteria to extract milk proteins specifically from whey protein because it is a main component of people’s daily diets and is consumed across the globe in both its pure and processed form.

Milk is a rich source of protein and peptides. It contains 3.3% protein, which is mainly divided into casein and whey protein. During the processing of milk into cheese, most of the casein is converted into cheese, which is used as an ingredient in people’s daily lives; the whey protein is released as a liquid in the cheese-making process. Whey is a by-product of cheese that is often drained out in the form of cheese waste. If whey composition is analyzed spectroscopically, it can be seen to contain a variety of protein and bioactive molecules, such as 20% entire milk proteins; 5% milk lactose; and 0.1–10% fats, minerals, and salts [9]. α-lactalbumin and β-lactoglobulin (1.3 g/L and 3.3 g/L. respectively) are the major components of whey protein. α-lactalbumin is the second most abundant globular protein source in whey and is composed of 123 amino acids with a high affinity for metals and ions; bovine serum albumin (0.3 g/L), bovine lactoferrin (0.1 g/L), immunoglobulins (0.5–1 g/L), lactoperoxidase (0.03 g/L), and proteinaceous glycophosphopeptide (1.2 g/L TMP) are minor components found in whey. Glycophosphopeptide (GMP) is a part of casein and seeps into whey protein during the cheese-making process. It consists of 64 amino acids and lacks the aromatic amino acids Phe, Tyr, and Trp. It is used as a prebiotic and an anti-inflammatory product [65,66].

Whey protein is an aggregate of the bioactive peptides that are encoded in the parent protein. These bioactive microelements can be derived from their parent proteins using different techniques, e.g., with enzymes such as pepsin, chymotrypsin, and trypsin; microbial enzymes (Alcalase TM, thermolysin, Flavourzyme TM, and proteinase); and fermentation. Trypsin is considered the best commercial enzyme to hydrolyze whey protein into bioactive peptides [67], while for fermentation, bacterial cultures, especially *LAB*, are used to hydrolyze protein into peptides; however, this technique is suitable only for small-scale production and not on an industrial scale [68]. In the previous literature, AMPs such as lactoferricin, lactoferrin B, lactoferrin C, and lactoferrin M have been purified from whey protein. These peptides exhibit pronounced antimicrobial activity against Gram-positive and Gram-negative bacteria [69].

**Figure 3:**
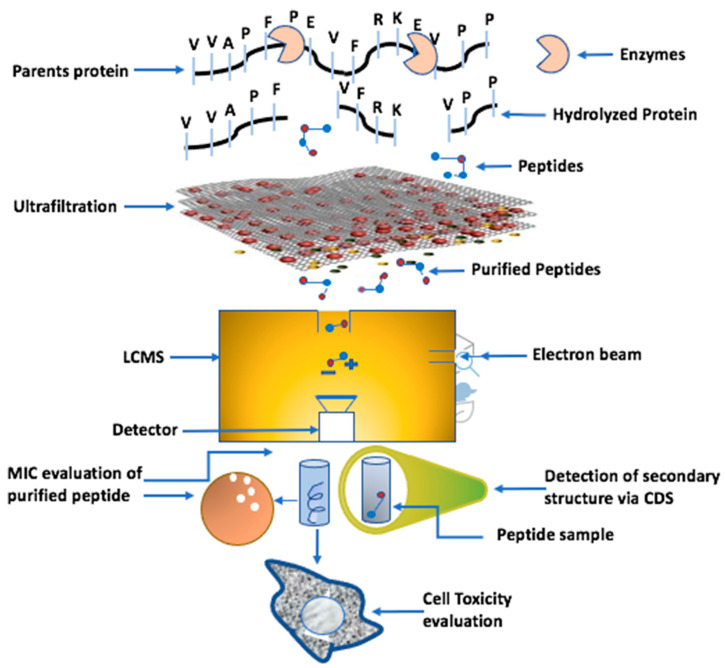
Shows the systemic pathway of purifying AMP from parent protein. LCMS, CSD, and MIC stand for liquid chromatography mass spectrometry, circular dichroism spectroscopy, and minimum inhibitory concentration, respectively.

The fermentation of whey proteins with *LAB* is considered the best way to hydrolyze milk protein into smaller peptides because *LAB* are equipped with extracellular serine protease, peptidases, and transport enzymes. Extracellular proteases hydrolyze proteins into peptides, and these peptides are transported into the intracellular peptidases, where they further hydrolyze into smaller peptides and are finally transported to extracellular spaces. The proteolytic systems of *LAB* are described in detail with many endopeptidases, aminopeptidases, tripeptidases, and dipeptidases. These *LAB* are used in the dairy industry to produce bioactive peptides from milk proteins, and these bioactive peptides are categorized as AMPs. The complete process of producing an AMP is depicted in Figure 3.

By documenting the latest research, we have found that whey protein is hydrolyzed with rennet enzyme by optimizing it to pH 3. Whey proteins are dissociated into AMP lactoferrin f(20–30), which has the ability to target and kill *Escherichia coli.* Lactoferrin attacks pathogenic bacteria by targeting the cell wall and causes membrane disruption [70]. Most of the research work related to evaluating the antimicrobial potency of lactoferrin and its derivates was documented in the first decade of the 21st century.

Different enzymes were used to produce lactoferrin fragments, e.g., the hydrolysis of whey protein with pepsin produced lactoferricin B and C, and pepsin and chymosin produced lactoferricin B, while some synthetic enzymes produced lactoferricin B, C, M, kaliocin-1, and lactoferrampin.

Apart from using enzymes for the purpose of protein hydrolysis and producing AMPs, different strains of LAB such as *Lactobacillus bulgaricus*, *Lactobacillus helveticus*, *Lactobacillus casei*, *Lactococcus lactis* were also used to hydrolyze dairy protein into bioactive peptides [69]. Goat whey was co-cultured with Alcalase TM, and AMP-like SEC-F2 and SEC-F3 were purified. These AMPs were targeted against *Escherichia coli* and *Bacillus cereus*. The exact structure and the nature of AMPs were not discussed in the findings [71]. In 2013, a study reported the production of novel AMPs from β-lactoglobulin (hydrolyzed with pepsin) that contained bactericidal effects against Gram-positive bacteria. After that, most of the focus was diverted to the production of angiotensin-converting enzyme (ACE) inhibitory peptides from whey protein [72].

Most of the bioactive peptides, such as α- Lactorphin, β-lactorphin, β-lactotensin, and serorphin, were extracted from hydrolyzed whey protein and tested against ACE inhibitory, immunomodulatory, and antioxidant activity [73]. These peptides with pronounced bioactivity can be tested against foodborne pathogens in the future.

## 7. Conclusions and Future Directions

AMPs are the answer to increasing antibiotic resistance. Researchers are trying to find nontoxic and safe-to-use AMPs, but the main problem starts with the production process for AMPs. After reviewing the data trends for post-2020, we have concluded that some studies are only confined to the production and evaluation of purified or prepared AMPs rather than being concerned with the designing of complete research plans for producing, purifying, and evaluating the efficiency of peptides as well as for evaluating the potency of those AMPs related to cytotoxicity and stability in the gut environment. The main thing that remains unknown is the targeting mechanism of AMPs. The use of scanning electron microscopy followed by transmission electron microscopy can explain the AMP targeting mechanism in most of the studies with logical literature findings and diagrams. However, further exploration is required in order to understand the exact mechanism more fully, which will help with designing potent AMPs in the future.

*Escherichia coli* and *Staphylococcus aureus* have been used as target pathogens against natural and synthesized AMPs, and some peptides have been reported to be cytotoxic; these need further modification to make them safe for therapeutic use.

The use of AMPs from whey proteins such as lactoferricins needs to be explored in future studies; most of the recent research is related to the production of the ACE inhibitory, immunomodulatory, and antioxidant activity of whey peptides. There is a need to move the research focus toward the production of novel active AMPs resulting from the hydrolyzing of α-lactalbumin and β-lactoglobulin.

## Data Availability

No new data were created or analyzed in this study. Data sharing is not applicable to this article.
